# lncRNA/miR-29c-Mediated High Expression of LOX Can Influence the Immune Status and Chemosensitivity and Can Forecast the Poor Prognosis of Gastric Cancer

**DOI:** 10.3389/fcell.2021.760470

**Published:** 2022-01-03

**Authors:** Aitao Nai, Huihui Zeng, Qiong Wu, Zirui He, Shuwen Zeng, Shoaib Bashir, Feng Ma, Jie He, Wei Wan, Meng Xu

**Affiliations:** ^1^ Department of Oncology, The First Affiliated Hospital of Jinan University, Guangzhou, China; ^2^ Department of Oncology, The First Affiliated Hospital of Bengbu Medical College, Bengbu, China; ^3^ Key Laboratory of Brain Science Research and Transformation in Tropical Environment of Hainan Province, Hainan Medical University, Haikou, China

**Keywords:** gastric cancer, lysyl oxidase (LOX), prognosis, immune microenvironment, immunotherapy, drug sensitivity

## Abstract

Gastric carcinoma is the fourth most prevalent cause of cancer-related deaths worldwide because of dismal prognosis and few therapeutic options. Accumulated studies have indicated that targeting lysyl oxidase (LOX) family members may serve as an anticancer strategy. Nevertheless, the specific mechanisms of LOX in stomach carcinoma are still unclear. In this study, we demonstrated that LOX is significantly different in 13 types of cancers and may act as a potential therapeutic target, especially in stomach carcinoma. Moreover, overexpression of LOX in gastric carcinoma was validated by multiple databases and contributed to the poor overall survival (OS), progression-free survival (PFS) and post-progression survival (PPS) of stomach adenocarcinoma (STAD) patients. Next, based on the ceRNA hypothesis, the HIF1A-AS2/RP11-366L20.2-miR-29c axis was characterized as the upstream regulatory mechanism of LOX gene overexpression in gastric cancer by combining correlation analysis, expression analysis, and survival analysis. Finally, we illustrated that LOX gene overexpression leads to dismal prognosis of gastric cancer, perhaps through promoting M2 macrophage polarization and tumor immune escape and enhancing drug resistance of tumor cells to chemotherapeutic drugs. Our research demonstrate that LOX may be potentially applied as a novel prognostic marker and targeting inhibition of LOX holds promise as a treatment strategy for gastric cancer.

## Introduction

Gastric cancer (GC) is a malignant tumor with relatively high morbidity and is the fourth most prevalent cause of cancer-related deaths worldwide and the second most prevalent cause of cancer-related deaths in China (Sung et al., 2021; Wu et al., 2020), which resulted in a considerable health burden. In spite of emerging progress in medical therapy and technology, the 5-year overall survival rate of patients with GC remains not satisfactory (Zhu et al., 2020). These features, such as the lack of early diagnostic techniques and effective treatment strategies, may account for the short survival in gastric cancer (Huang et al., 2015). Therefore, identification of new disease mechanisms to seek novel prognostic biomarkers and therapeutic targets is of profound significance.

Lysyl oxidase (LOX) is a well-characterized member of this family and is also defined as an amine oxidase involved in extracellular matrix remodeling (Han et al., 2019a). Accumulated studies suggest that LOX is closely related to tumor cell proliferation, migration, invasion, and metastasis (Ye et al., 2020; Li et al., 2019) and is perhaps a potential molecular target for tumor therapy (Setargew et al., 2021). Several publications demonstrate that the mRNA and protein expression of LOX is observably overexpressed in stomach cancer (Han et al., 2019b; Peng et al., 2018; Zhang et al., 2013). Nevertheless, the detailed biological function of LOX in gastric cancer is still poorly understood.

LOX family members contribute to the establishment and maturation of the tumor microenvironment (Tenti and Vannucci, 2020; Lin et al., 2020). The tumor microenvironment is composed of a variety of cell types, such as stromal cells, cancer cells, and immune cells (Schurch et al., 2020). The connection between LOX expression and tumor immune infiltration in stomach adenocarcinoma (STAD) is, however, not yet understood. Furthermore, previous research showed that targeting LOX family members is a therapeutic strategy for overall survival (OS) management through improving the efficacy of the current chemotherapies (Matsuoka et al., 2020). Then, is there a correlation between LOX expression and chemotherapeutic drug sensitivity of gastric carcinoma?

In this study, we first investigated the mRNA expression of LOX between tumor and adjacent non-cancer tissues in multiple databases and further investigated the correlation of LOX with clinical characteristics of STAD. Then, we performed the prognostic value of high LOX expression in STAD and also predicted potential miRNAs and lncRNAs to illustrate the possible mechanisms responsible for up-regulation of LOX in STAD. Ultimately, we analyzed the correlation of LOX expression with tumor microenvironment, immune cell infiltration, markers of immune cells, immune checkpoints, and chemotherapeutic drug sensitivity to elucidate the biological functions of high LOX expression in gastric cancer. Our study demonstrates that the HIF1A-AS2/RP11-366L20.2-miR-29c-mediated high expression of LOX can influence the immune status and chemosensitivity and can forecast the poor prognosis of gastric cancer.

## Materials and Methods

### Data and Sources

Gene expression data of LOX were obtained from the UALCAN database (http://ualcan.path.uab.edu/), Gene Expression Profiling Interactive Analysis (GEPIA, http://gepia.cancer-pku.cn/detail.php), University of California Santa Cruz (UCSC) Xena dataset (http://xena.ucsc.edu/), and Gene Expression Omnibus dataset (GEO, https://www.ncbi.nlm.nih.gov/geo/). The clinical and survival data of gastric cancer were obtained from Kaplan–Meier plotter (http://kmplot.com/analysis/), UCSC Xena dataset, and GEO. The chemotherapy sensitivity data was accessed through the CellMiner database (https://discover.nci.nih.gov/cellminer/). Moreover, the expression data of miRNAs and lncRNAs were obtained from UCSC Xena dataset.

### Survival Analysis

We analyzed the relationship of LOX with the OS, progression-free survival (PFS), and post-progression survival (PPS) of STAD patients in Kaplan–Meier plotter database. The Kaplan–Meier plotter database includes six STAD subsets (GSE14210, GSE15459, GSE22377, GSE29272, GSE51105, and GSE62254). All samples were distinguished into two groups according to the median. The log-rank test was applied to evaluate survival differences between groups. Meanwhile, survival analysis in The Cancer Genome Atlas (TCGA) and GSE84437 dataset was performed through “survminer” and “survival” packages in R software.

### Candidate miRNA and lncRNA Forecast

Upstream binding miRNAs of LOX was forecasted through the ENCORI pan-cancer analysis platform (http://starbase.sysu.edu.cn/panCancer.php), which consists of PITA, RNA22, miRmap, microT, miRanda, PicTar, and TargetScan programs. Only the forecasted miRNAs that simultaneously presented in five or more programs as described above were considered for following analyses (Lou et al., 2021) (Supplementary Table S1). The correlation between miRNAs and LOX in STAD was analyzed by the Spearman method. Upstream binding lncRNAs of miRNA was forecasted in ENCORI (Supplementary Table S2). The correlation between lncRNAs and miRNA-29c or LOX in STAD was also analyzed by the Spearman method. The Kaplan–Meier method was applied to assess survival differences of lncRNAs or miRNAs. Moreover, the expression difference of lncRNAs or miRNAs was evaluated by unpaired *t*-test.

### Stromal and Immune Score Calculation and Immune Infiltration Analysis

Immune score, stromal score, and ESTIMATE score of STAD patients were analyzed by the “Estimate” R package based on TCGA and Gene Expression Omnibus (GEO) gene expression profiles. The prognostic significance of immune cells and the correlation between LOX and immune cells (B cells, CD4^+^ T cells, CD8^+^ T cells, neutrophils, myeloid dendritic cell, and macrophages), markers of immune cells, and immune checkpoints in gastric carcinoma were obtained from the Tumor Immune Estimation Resource (TIMER, https://cistrome.shinyapps.io/timer/). Furthermore, CIBERSORT was used to analyze the immune cell fractions of STAD samples from TCGA and GEO.

### Gene Set Enrichment Analysis

The 375 STAD patients in TCGA and 433 patients in GSE84437 were respectively divided into a high-LOX-expression group and a low-LOX-expression group based on its median expression values. Gene set enrichment analysis (GSEA) was conducted on the above datasets to clarify the potential KEGG pathways (c2.cp.kegg.v7.4) and HALLMARKS terms (h.all.v7.4) between groups with high and low LOX expression. Only gene sets with adjusted *p* value <0.05 and |NES| > 1 were considered statistically significant.

### The Correlation Analysis Between LOX and Chemotherapy Sensitivity

The NCI-60, a panel of 60 distinct human carcinoma cell lines from nine diverse kinds of cancers, was applied to analyze chemotherapy sensitivity in CellMiner database (Lin et al., 2021). After we removed the cancer cell lines with more than 80% missing data, 59 cancer cell lines and 792 drugs that received FDA approval or on clinical trials were included in the correlation analyses (Supplementary Table S3). The correlation between LOX gene expression and drug sensitivity was assessed by Spearman correlation analysis.

### Statistical Analysis

Paired *t*-test or unpaired *t*-test was performed to compare the difference in LOX gene expression levels between the normal and tumor groups in R software (version 4.0.3). The gene expression difference of various clinicopathological parameters was compared by the unpaired *t*-test or Kruskal–Wallis test. A *p* value <0.05 indicated statistical significance.

## Results

### LOX is Overexpressed in Gastric Cancer

**FIGURE 1:**
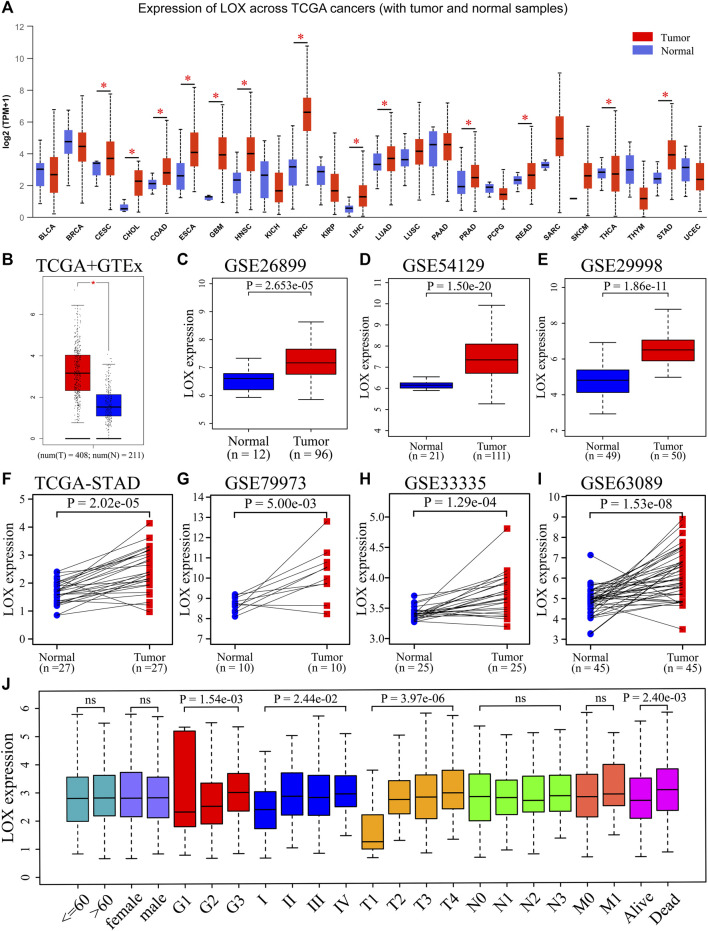
The mRNA of lysyl oxidase (LOX) is overexpressed in gastric cancer. **(A)** Pan-cancer analysis of LOX mRNA expression across cancers from UALCAN dataset. **(B)** LOX mRNA expression levels in stomach adenocarcinoma (STAD) and normal tissues in The Cancer Genome Atlas (TCGA) + GTEx dataset. **(C–E)** LOX mRNA expression levels in STAD and normal tissues in GSE26899 **(C)**, GSE54129 **(D)**, and GSE29998 **(E)** datasets. **(F)** LOX mRNA expression in STAD and matched normal tissues in TCGA dataset. **(G–I)** LOX mRNA expression in STAD and matched normal tissues in GSE79973 **(G)**, GSE33335 **(H)**, and GSE63089 **(I)** datasets. **(J)** The correlation between LOX mRNA expression and clinicopathological parameters of STAD patients from TCGA database. ns, no significance; ^*^ *p* < 0.05.

To explore the expression of LOX in multiple malignancies, the expression of LOX in various cancer types was analyzed by the UALCAN database (Figure 1A). The results revealed that LOX mRNA expression was considerably increased in the vast majority of human cancers compared with non-cancer tissues, such as cervical squamous cell carcinoma, endocervical adenocarcinoma, cholangiocarcinoma, colon adenocarcinoma, esophageal carcinoma, glioblastoma multiforme, head and neck squamous cell carcinoma, kidney renal clear cell carcinoma, liver hepatocellular carcinoma, lung adenocarcinoma, prostate adenocarcinoma, rectal adenocarcinoma, and stomach adenocarcinoma, while it was markedly lower in thyroid carcinoma. These results indicate that LOX has a considerably different expression in most malignancies, and it may function as a potential oncogene, particularly in gastric carcinoma.

To further authenticate the results in gastric carcinoma, the quantitative evaluation of LOX expression level was examined by an analysis of multiple databases. The results indicated that the mRNA expression of LOX was markedly higher in gastric carcinoma than that in normal tissues in TCGA + GTEx (Figure 1B), GSE26899 (Figure 1C), GSE54129 (Figure 1D), and GSE29998 (Figure 1E) databases. Data from gastric cancer and matched adjacent normal tissues further verified these results in TCGA (Figure 1F), GSE79973 (Figure 1G), GSE33335 (Figure 1H), and GSE63089 (Figure 1I) databases.

Besides, we examined LOX expression on the basis of patients’ multiple clinical features in order to elucidate the relevance between LOX expression level and clinical characteristics of STAD. An analysis of the TCGA data showed that LOX mRNA expression was positively related to histological grade, TNM stage, T stage, and patient survival status, but not with age, gender, nodal stage, and distant metastasis (Figure 1J). These data indicate that the overexpression of LOX may serve a crucial role in the tumorigenesis and development of STAD.

### Overexpression of LOX is Connected With Worse Prognosis of STAD

**FIGURE 2:**
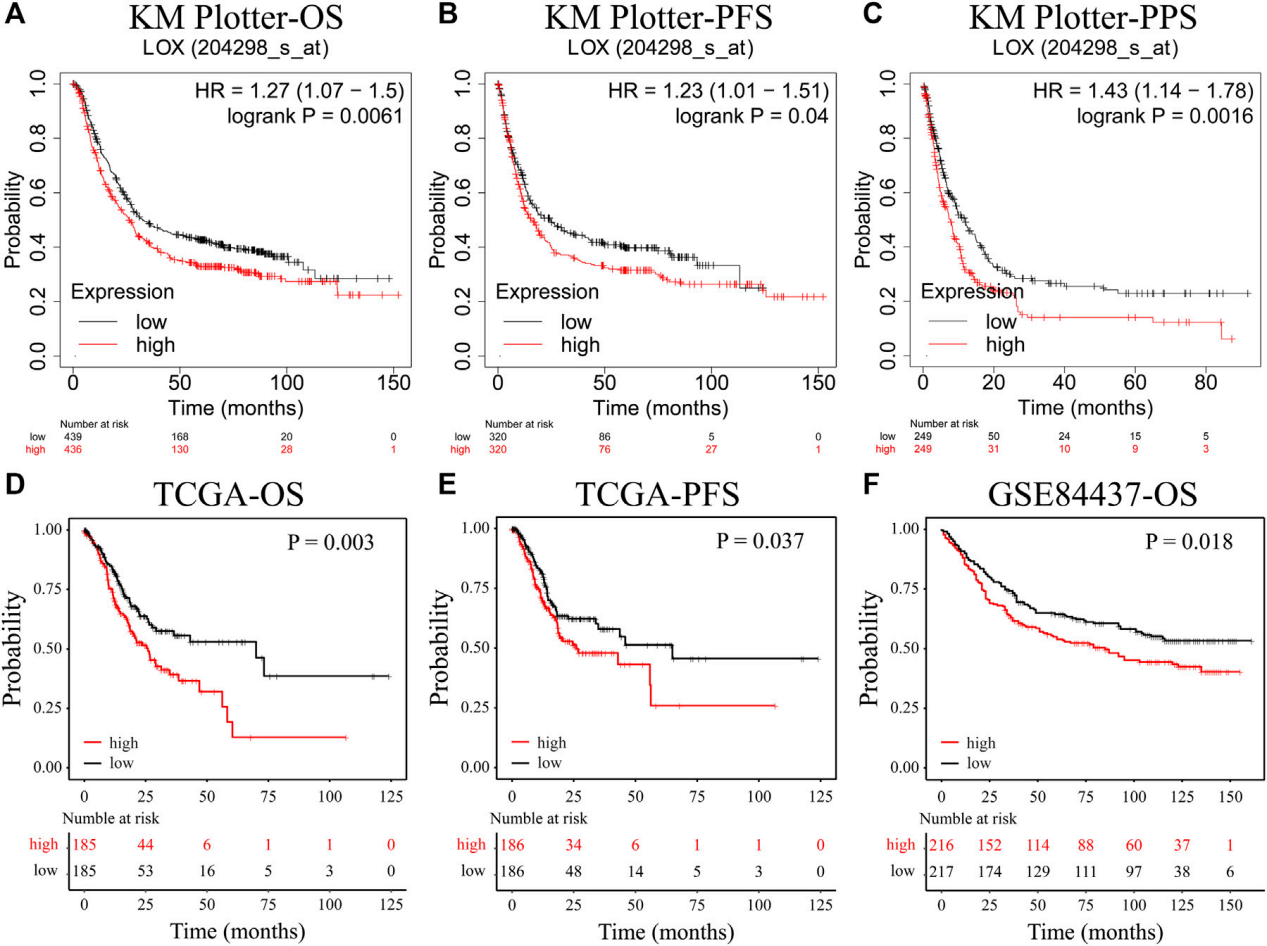
The overexpression of LOX is correlated with poor prognosis of STAD. **(A)** The overall survival curves of LOX in STAD patients (Kaplan–Meier plotter). **(B)** The progression-free survival curves of LOX in STAD patients (Kaplan–Meier plotter). **(C)** The post-progression survival curves of LOX in STAD patients (Kaplan–Meier plotter). **(D)** The overall survival curves of LOX in STAD patients (TCGA). **(E)** The progression-free survival curves of LOX in STAD patients (TCGA). **(F)** The overall survival curves of LOX in STAD patients (GSE84437).

The previous results indicated that those patients who died showed a markedly higher LOX gene expression; hence, we subsequently explored the relevance between LOX expression and prognosis in STAD. Kaplan–Meier plotter database was applied to draw the OS, PFS, and PPS curves. All the patients were categorized into high- and low-expression groups according to the mean expression value of LOX. It was worth noting that the overexpression of LOX contributed to the poor OS (Figure 2A), PFS (Figure 2B), and PPS (Figure 2C) of STAD patients. Furthermore, the overexpression of LOX, which predicts worse OS and PFS, was also verified in TCGA database (Figures 2D,E) and GSE84437 database (Figure 2F).

### Forecasting the Upstream miRNAs of LOX

**FIGURE 3:**
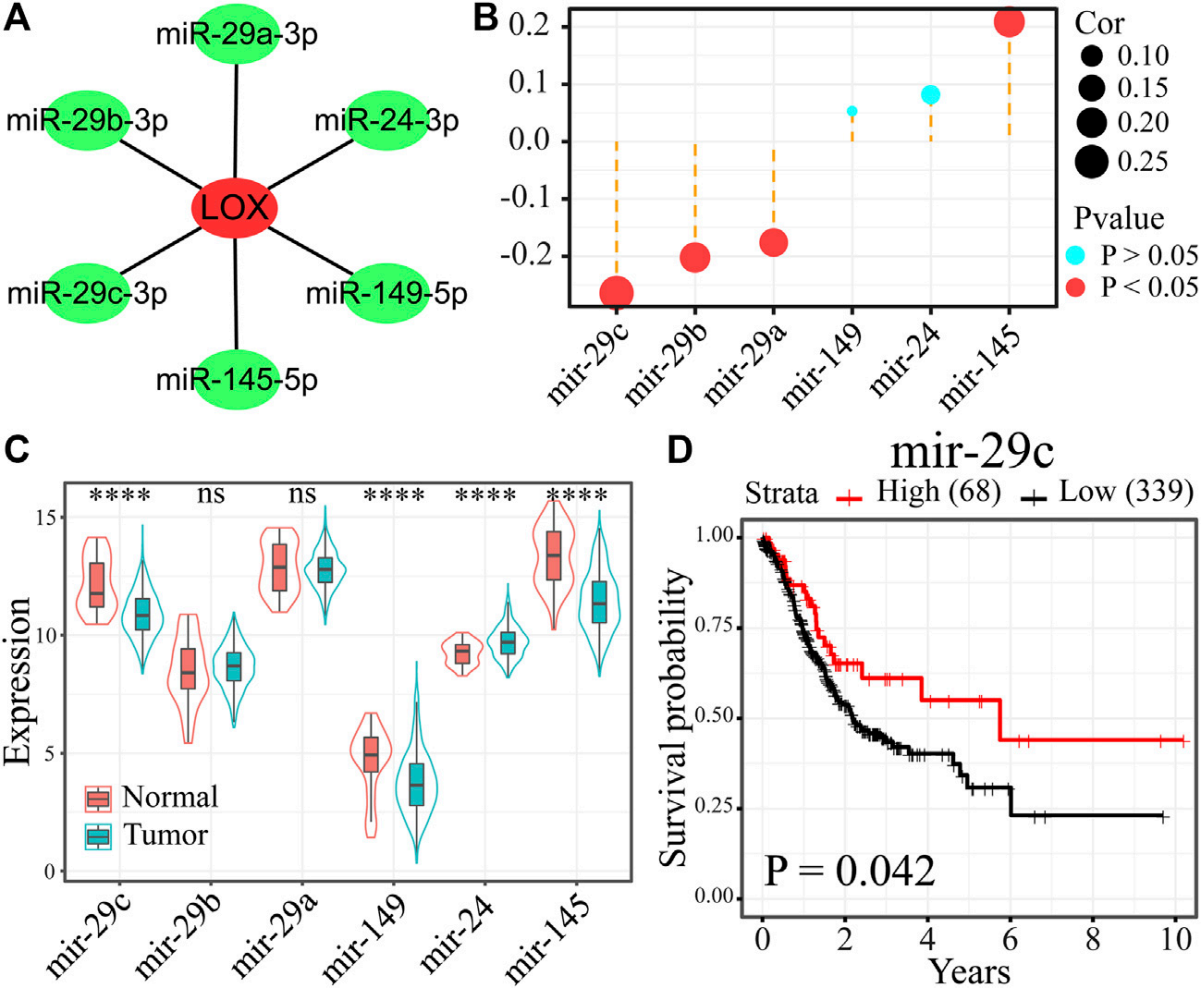
miRNA-29c might be the most probable upstream regulatory miRNA of LOX in gastric cancer. **(A)** Six potential upstream miRNA candidates of LOX were presented using Cytoscape software. **(B)** The relevance analysis between six miRNAs and LOX expression values in 372 STAD samples. **(C)** The expression of six miRNAs in 436 STAD samples and 41 non-cancer samples. **(D)** The survival curves of miRNA-29c in STAD patients (Kaplan–Meier plotter). ^****^ *p* < 0.0001.

Non-coding RNAs are a class of RNA transcripts that are not translated into proteins but are related to the regulation of gene expression (Levick and Widiapradja, 2020). Therefore, we first predicted upstream miRNAs that could be interacting with LOX in ENCORI database. We then selected six miRNA candidates that possessed LOX mRNA-binding sites and commonly appeared more than five in PITA, RNA22, miRmap, microT, miRanda, PicTar, and TargetScan programs (Figure 3A; Supplementary Table S1). Next, we carried out the correlation analysis between LOX with above-six miRNAs in 372 STAD samples of TCGA database. Based on the nature of miRNA function, miRNA and target genes are normally negatively regulated, and only miRNA-29c, miRNA-29b, and miRNA-29a meet this condition (Figure 3B). Further expression analysis demonstrated that only miRNA-29c among the above three miRNAs was differentially expressed (Figure 3C). Meanwhile, the low expression of miRNA-29c was positively related with poor prognosis of STAD patients (Figure 3D). All the results indicate that miRNA-29c is perhaps the most probable upstream regulatory miRNA of LOX in gastric cancer.

### Forecasting the Upstream lncRNAs of miRNA-29c

**FIGURE 4:**
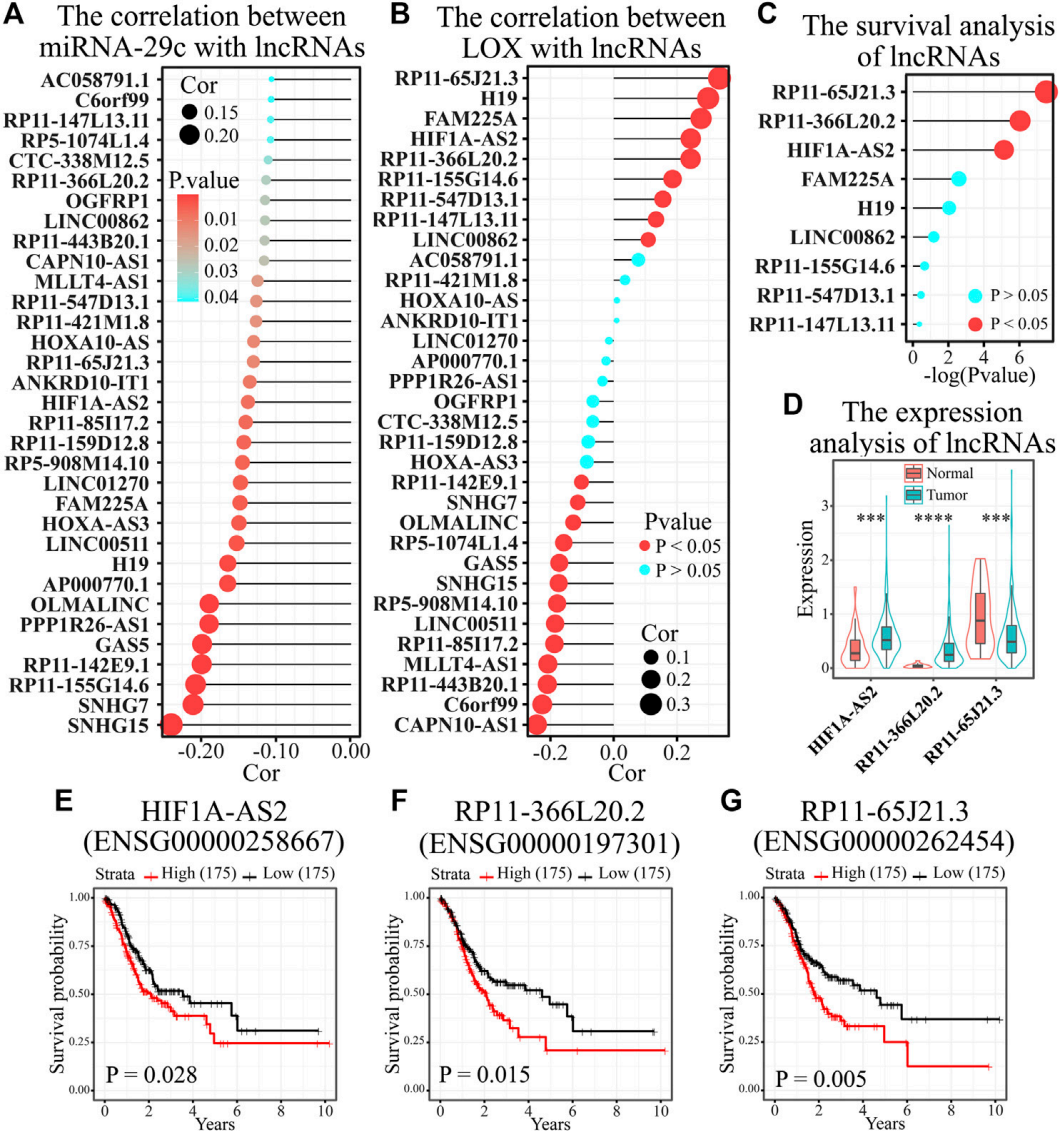
HIF1A-AS2 and RP11-366L20.2 were considered as the most possible upstream regulatory lncRNAs of the miRNA-29c/LOX axis in gastric cancer. **(A)** Thirty-three of 298 potential upstream lncRNA candidates were negatively correlated with miRNA-29c in gastric cancer. **(B)** The relevance analysis between 33 lncRNAs and LOX in 372 STAD samples. **(C)** The survival analysis of nine lncRNAs positively correlated with LOX in gastric cancer. **(D)** The expression analysis of nine lncRNAs in 375 STAD samples and 32 non-cancer samples. **(E–G)** The survival curves of HIF1A-AS2 **(E)**, RP11-366L20.2 **(F)**, and RP11-65J21.3 **(G)** in gastric cancer. ^***^ *p* < 0.001 and ^****^ *p* < 0.0001.

Next, we forecasted the possible upstream regulatory lncRNAs of miRNA-29c by ENCORI database. A total of 298 potential lncRNA candidates with miRNA-29c-binding sites were predicted (Supplementary Table S2). Considering the ceRNA hypothesis that the relevance between lncRNA and miRNA was negative while the relevance between lncRNA and mRNA was positive (Lei et al., 2021), we firstly performed a correlation analysis between miRNA-29c and the 298 lncRNAs in 372 STAD samples of TCGA databases. Among them, only 33 lncRNA–miRNA pairs were inversely related (Figure 4A). Then, the correlation analysis between LOX and 33 lncRNAs showed that nine lncRNAs were positively related with LOX in STAD samples (Figure 4B). Subsequently, we found that HIF1A-AS2, RP11-366L20.2, and RP11-65J21.3 have both differential expression and differential survival (Figures 4C–G). However, RP11-65J21.3 has low expression in STAD samples, and its low expression showed a satisfactory prognosis, which is inconsistent with the ceRNA hypothesis. Therefore, by combination of correlation analysis, expression analysis, and survival analysis, HIF1A-AS2 and RP11-366L20.2 were considered as the most possible upstream regulatory lncRNAs of the miR-29c/LOX axis in gastric cancer.

### LOX Is Involved in Immune Cell Infiltration in STAD

**FIGURE 5:**
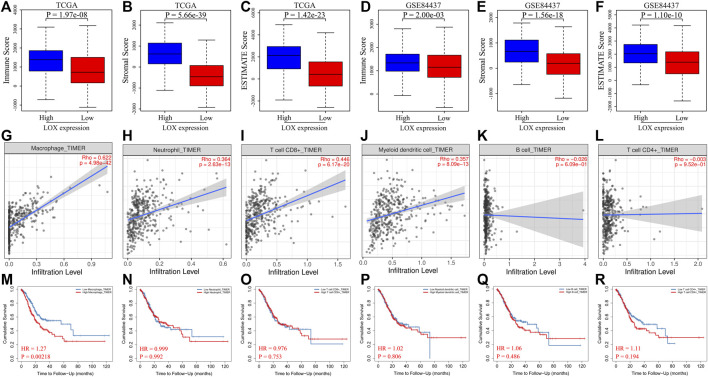
The overexpression of LOX is related to immune cell infiltration of STAD. **(A–C)** Comparison of immune score **(A)**, stromal score **(B)**, and ESTIMATE score **(C)** between the high- and low-LOX-expression groups of STAD in TCGA database. **(D–F)** Comparison of immune score **(D)**, stromal score **(E)**, and ESTIMATE score **(F)** between the high- and low-LOX-expression groups of STAD in GSE84437 database. **(G–L)** The correlation between LOX expression and macrophages **(G)**, neutrophils **(H)**, CD8^+^ T cells **(I)**, myeloid dendritic cells **(J)**, B cells **(K)**, and CD4^+^ T cells **(L)** of gastric cancer in TIMER database. **(M–R)** The overall survival analysis for macrophages **(M)**, neutrophils **(N)**, CD8^+^ T cells **(O)**, myeloid dendritic cells **(P)**, B cells **(Q)**, and CD4^+^ T cells **(R)** of gastric cancer in TIMER database.

Research showed that LOX is strongly associated with the establishment and maturation of the tumor microenvironment. We speculated whether the overexpression of LOX caused an alteration of the tumor microenvironment in gastric cancer. Hence, immune, stromal, and ESTIMATE scores were calculated by the ESTIMATE algorithm according to gene expression values of TCGA and GSE84437 databases. The results showed that immune, stromal, and ESTIMATE scores in the high-LOX-expression group were remarkably higher compared to the low-LOX-expression group in TCGA (Figures 5A–C) and GSE84437 (Figures 5D–F) databases, which supports our guess that the overexpression of LOX indeed modifies the tumor microenvironment in gastric cancer. To further illustrate the correlation between LOX and tumor immune microenvironment, we evaluated the relationship of LOX and six types of immune-infiltrating cells in TIMER database. As shown in Figures 5G–L, LOX expression was clearly positively related with macrophage, neutrophil, CD8^+^ T cells, and myeloid dendritic cells, while no correlation emerged with B cell and CD4^+^ T cells. The survival analysis results indicated that only macrophage in six types of immune cells presented a markedly differential survival in TIMER database (Figures 5M–R). These findings demonstrate that the overexpression of LOX may influence immune cell infiltration, especially macrophages, which results in the worse prognosis of STAD patients.

**FIGURE 6:**
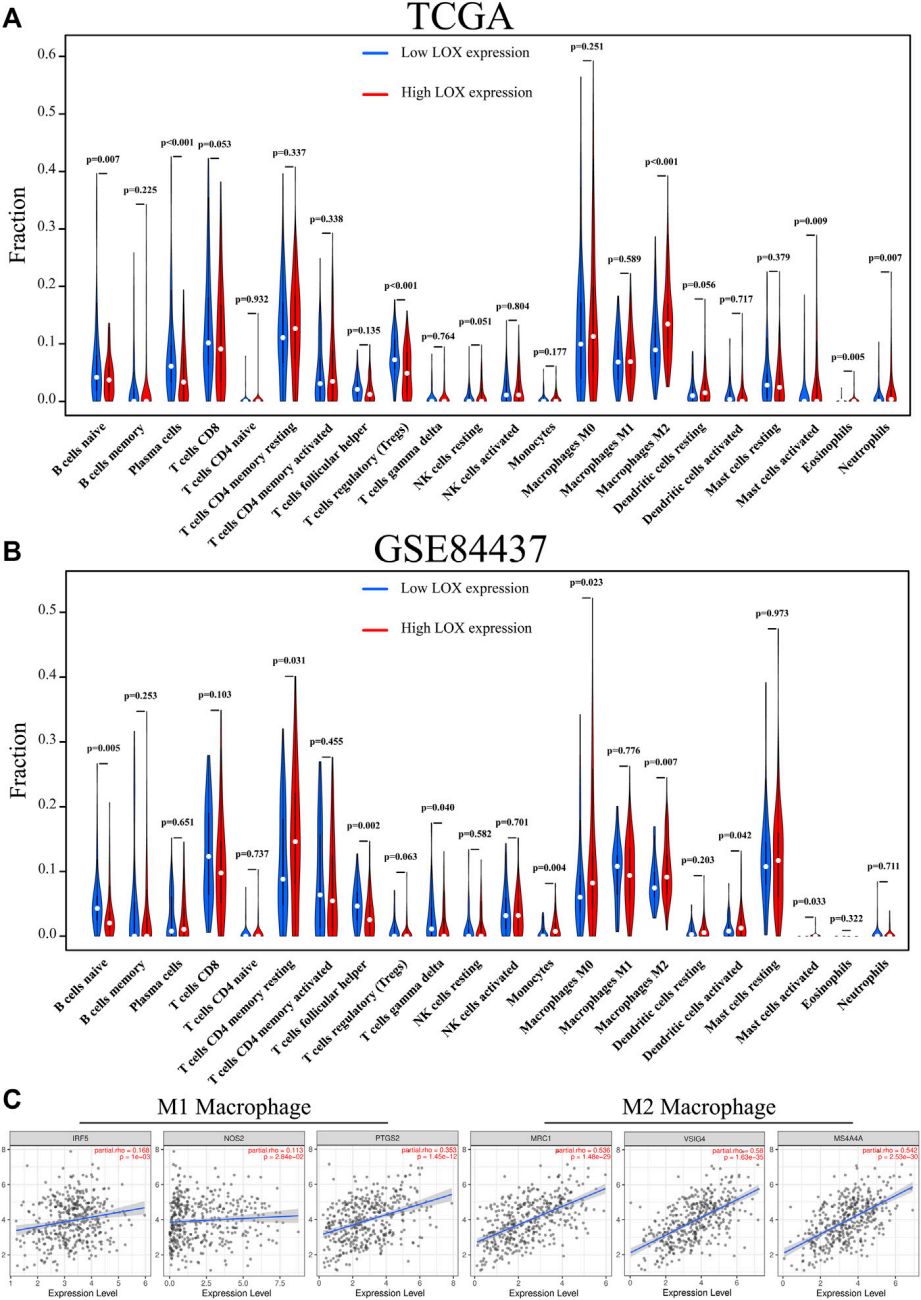
The overexpression of LOX can promote macrophage polarization towards M2 phenotype. **(A)** Proportions of the 22 tumor-infiltrating immune cell types in the high- and low-LOX-expression groups in TCGA database. **(B)** Proportions of the 22 tumor-infiltrating immune cell types in the high- and low-LOX-expression groups in GSE84437 database. **(C)** The correlation between LOX expression and markers of M1 and M2 macrophages.

Tumor-associated macrophages can be generally categorized as tumor-suppressive M1 and tumor-supportive M2 subtypes (Liao et al., 2019). Therefore, we further investigated the infiltration fraction of M1 macrophage and M2 macrophage in TCGA and GSE84437 databases. We observed that the infiltration fraction of M2 macrophage was remarkably higher in the high-LOX-expression group compared to the low-LOX-expression group in TCGA database (Figure 6A), which was also corroborated in GSE84437 database (Figure 6B). The correlation analysis results also revealed that the correlation between LOX and M2 macrophage markers is remarkably higher than that of M1 macrophage (Figure 6C). These results suggest that the overexpression of LOX can promote macrophage polarization towards the M2 phenotype.

**FIGURE 7:**
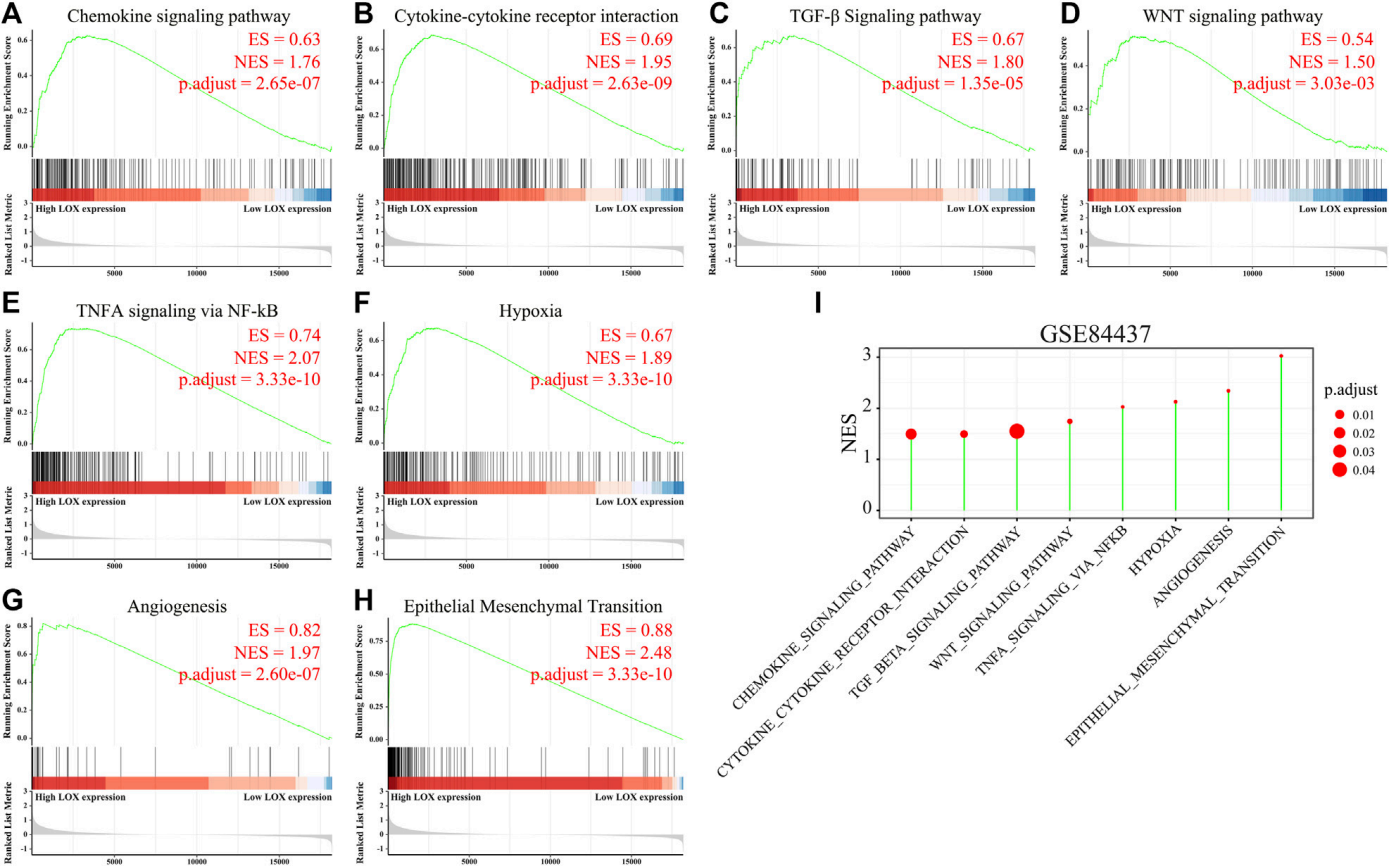
Many pathways associated with M2 macrophage were enriched in the high-LOX-expression group. **(A–H)** Gene set enrichment analysis (GSEA) of M2 macrophage-related enrichment gene sets in the STAD samples from TCGA database, including chemokine signaling pathway **(A)**, cytokine–cytokine receptor interaction **(B)**, TGF-β signaling pathway **(C)**, WNT signaling pathway **(D)**, TNFα signaling pathway **(E)**, hypoxia **(F)**, angiogenesis **(G)**, and epithelial–mesenchymal transition **(H)**. **(I)** GSEA analysis of M2 macrophage-related enrichment gene sets in the STAD samples from GSE84437 database.

To further elucidate the biological function of M2 macrophage in stomach cancer, GSEA was performed. Enrichment analysis results indicated that many signaling pathways associated with M2 macrophage in TCGA database, such as chemokine signaling pathway (Figure 7A), cytokine–cytokine receptor interaction (Figure 7B), TGF-β signaling pathway (Figure 7C), WNT signaling pathway (Figure 7D), TNFα signaling *via* NF-kB (Figure 7E), hypoxia (Figure 7F), angiogenesis (Figure 7G), and epithelial–mesenchymal transition (Figure 7H), were considered to be significantly activated in the high-LOX-expression group, which agreed with the results of the enrichment analysis in the GSE84437 database (Figure 7I). All these data together indicate that the overexpression of LOX potentially facilitates tumor progress and leads to a worse prognosis for patients with gastric cancer by promoting M2 macrophage polarization, which then activates a series of downstream cancer-promoting signaling pathways.

### The Relationship Between LOX and Chemosensitivity in STAD

**FIGURE 8:**
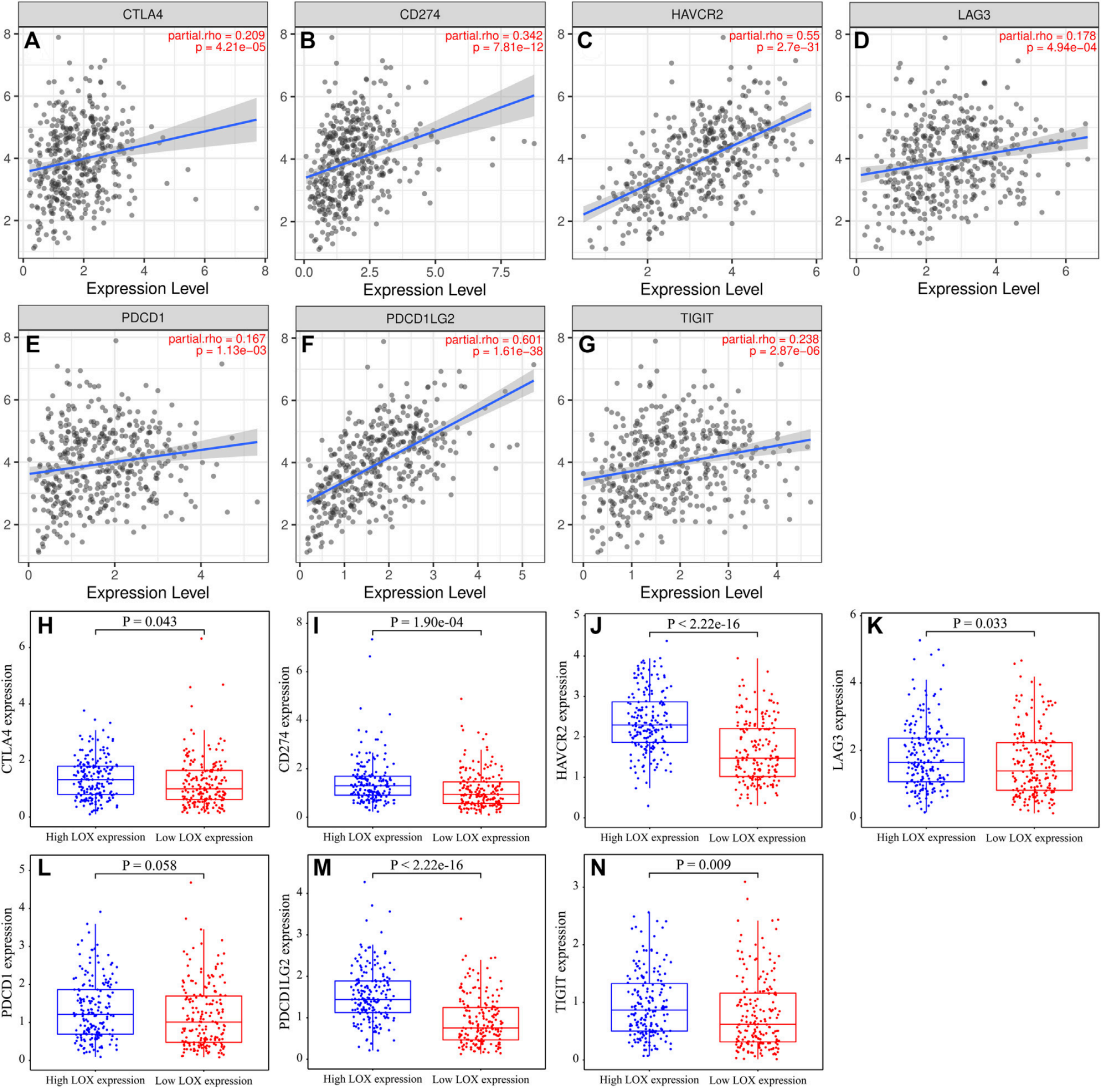
The overexpression of LOX was negatively correlated with chemotherapeutic drug sensitivity. **(A–H)** The correlation between LOX expression and chemotherapeutic drug sensitivity, including fluorouracil **(A)**, oxaliplatin **(B)**, docetaxel **(C)**, methotrexate **(D)**, tegafur **(E)**, and paclitaxel **(F)**.

In order to further ascertain the reason for the more undesirable prognosis of STAD patients with high LOX expression, the correlation between LOX expression and drug sensitivity was assessed by the Spearman method. We found that LOX expression was negatively correlated with the sensitivity of some commonly used chemotherapeutic drugs, such as fluorouracil (Figure 8A), oxaliplatin (Figure 8B), docetaxel (Figure 8C), methotrexate (Figure 8D), tegafur (Figure 8E), and paclitaxel (Figure 8F), which suggests that an enhanced drug resistance of tumor cells to multiple chemotherapeutic agents might be involved in high LOX expression-mediated poor prognosis of STAD patients.

### The Relationship Between LOX and Immune Checkpoints in STAD

**FIGURE 9:**
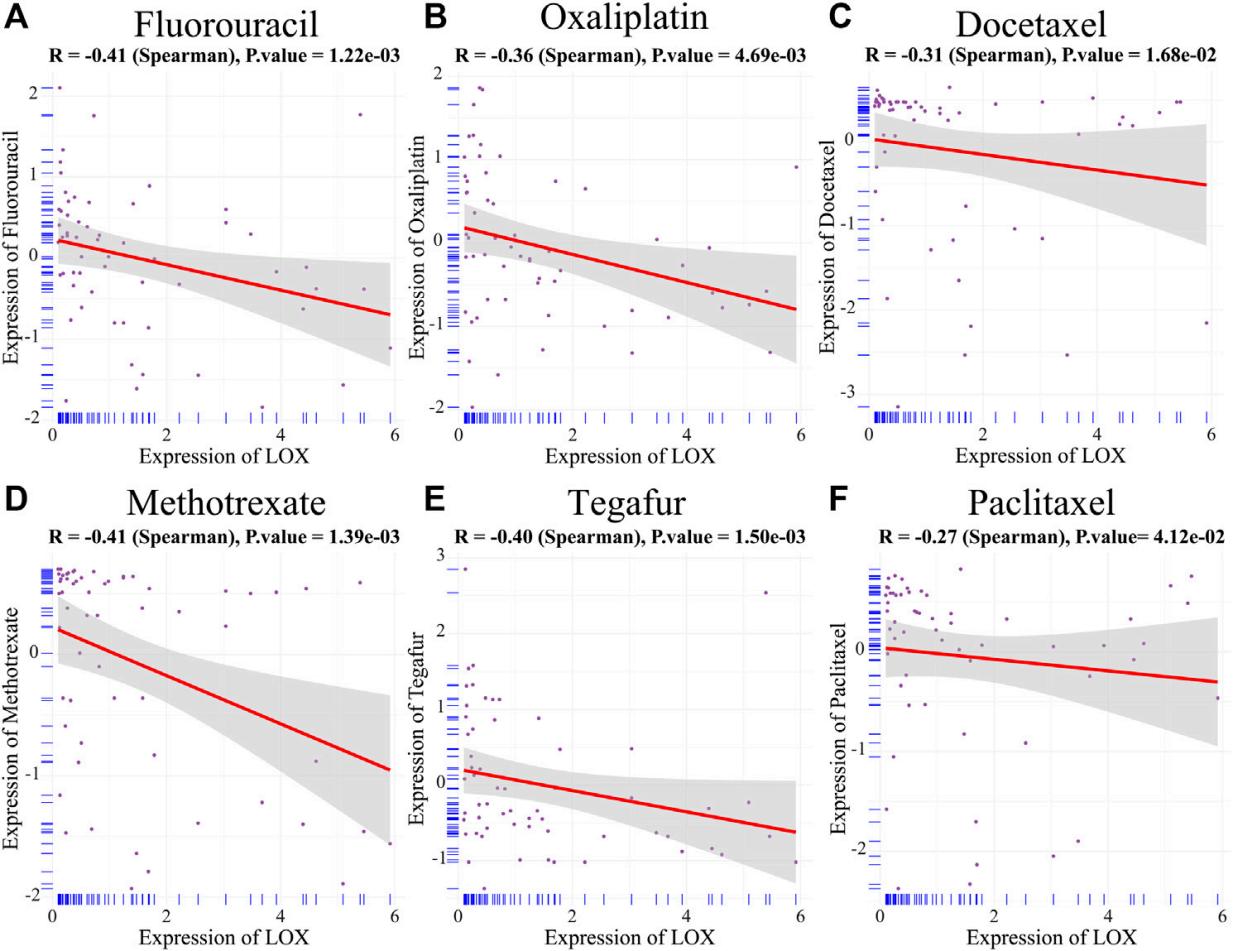
The overexpression of LOX was positively associated with immune checkpoints. **(A–G)** The relationship between LOX expression and immune checkpoints, including CTLA4 **(A)**, CD274 **(B)**, HAVCR2 **(C)**, LAG3 **(D)**, PDCD1 **(E)**, PDCD1LG2 **(F)**, and TIGIT **(G)** in TIMER database. **(H–N)** The expression of immune checkpoints in high LOX expression and low LOX expression in TCGA dataset.

In consideration of this concern that tumor cells can modify the tumor immune microenvironment by activating immune checkpoints (Lee et al., 2021), we therefore analyzed the correlations between LOX expression and several immune checkpoints. The results revealed that LOX expression was positively associated with CTLA4, CD274, HAVCR2, LAG3, PDCD1, PDCD1LG2, and TIGIT (Figures 9A–G). Besides, the seven immune checkpoints above except PDCD1 were all significantly up-regulated in the high-LOX-expression group (Figures 9H–N). The results above suggest that immune escape of cancer cells mediated by immune checkpoint may be an important prognostic factor of high LOX expression-mediated tumor progression.

## Discussion

Recently, numerous studies reported that targeting LOX family members may serve as an anticancer strategy (Setargew et al., 2021; Barker et al., 2012). In this study, we found that among 13 cancer types with differential expression of LOX, 12 tumor types were found with significantly overexpressed LOX, including gastric cancer. The result indicates that LOX may function as a potential oncogene and may be proposed as a promising potential molecular target for therapy in various types of cancers. In particular, further analysis based on multiple databases further supported the oncogenic effect of LOX in gastric carcinoma. Furthermore, we also demonstrated that the overexpression of LOX in gastric cancer was positively related to histological grade; TNM stage; T stage; and poor OS, PFS, and PPS. These results suggest that LOX perhaps acts as a possible prognostic biomarker in stomach cancer.

It is well known that the major biological functions of miRNAs are repressing the expression of their target genes (Wei et al., 2018). Therefore, in order to identify the upstream miRNA candidates that mediated LOX overexpression in gastric cancer, we predicted miRNAs that possibly target LOX in multiple target prediction programs, such as PITA, RNA22, miRmap, microT, miRanda, PicTar, and TargetScan programs. The integrative analysis combining correlation, differential expression, and survival analysis showed that miRNA-29c might be the best upstream miRNA candidate interacting with LOX in gastric cancer. Interestingly, multiple previous studies have demonstrated that miRNA-29c was remarkably down-regulated in gastric cancer compared with normal tissues (Vidal et al., 2016; Han et al., 2015; Jiang et al., 2019; Matsuo et al., 2013) and the overexpression of miRNA-29c inhibits cell proliferation (Yu et al., 2017) and migration (Wang et al., 2019). Furthermore, multiple studies also have demonstrated that the low expression of miRNA-29c indicates poor prognosis in gastric cancer (Chen et al., 2021; Y.; Han et al., 2019a). According to the above research and considering that five of the seven target prediction programs presented miRNA-29c and LOX-binding sites, we believe that miRNA-9c is the most appropriate upstream regulatory miRNA for LOX in gastric carcinoma.

We also predicted the upstream lncRNAs of miRNA-29c in gastric cancer. Based on the ceRNA hypothesis that the relevance between lncRNA and miRNA was negative, while the relevance between lncRNA and mRNA was positive (Zhou et al., 2020; Lei et al., 2021), nine lncRNAs were considered for following analyses. Further expression analysis and survival analysis showed that only HIF1A-AS2 and RP11-366L20.2 fulfilled our criteria that they are highly expressed in tumors and that their high expression indicates a worse prognosis. It was reported that HIF1A-AS2 is up-regulated in GC tumorous tissues, and its overexpression promotes gastric cancer cell proliferation and metastasis and indicates poor prognosis (Mu et al., 2021; Chen et al., 2015). RP11-366L20.2, also known as HMGA2-AS1, has been recognized as an oncogene in pancreatic cancer (Ros et al., 2019) and osteosarcoma (Rothzerg et al., 2021). However, there are few studies on the role of RP11-366L20.2 in gastric cancer, and it is worthy of further study. In summary, we suggest that the differential expression of the HIF1A-AS2/RP11-366L20.2-miRNA-29c axis is considered as one of the causes of high expression of LOX in gastric carcinoma.

As is known, the tumor microenvironment contains stromal cells and immune cells (Bottcher et al., 2018). LOX induces activation of tumor stromal cells and facilitates the development and progression of gastric carcinoma (Peng et al., 2018). However, the relevance of LOX and immune cells in gastric cancer still remains unclear. Our findings indicated that the stromal score in the high-LOX-expression group was remarkably increased, which seems to be consistent with what is reported in the above article (Peng et al., 2018). Interestingly, the immune score was also obviously improved in the high-LOX-expression group, which was validated by two independent datasets (TCGA and GSE84437). Further correlation analysis and survival analysis results indicated that tumor-associated macrophage infiltration probably contributes to the dismal prognosis of patients with high LOX expression in gastric carcinoma.

M1 macrophage can inhibit cancer growth, and M2 macrophage can accelerate it (Kim et al., 2019). Our analysis in TCGA and GSE84437 datasets demonstrated that the infiltration fraction of M2 macrophage was significantly higher in the high-LOX-expression group. The activation of WNT signaling pathway and hypoxia can promote M2 macrophage polarization (Yang et al., 2018; Laoui et al., 2014), which in turn can secrete WNT ligands to modulate WNT pathway and transcriptionally regulate HIF-1a by NF-kB (Cosin-Roger et al., 2019; Capece et al., 2013). Moreover, M2 macrophage also releases cytokines and chemokines, such as IL-6, TNFα, and CCL22 (Komohara et al., 2016), activates TGF-β signaling pathway (Yin et al., 2019), and promotes angiogenesis and epithelial–mesenchymal transition (Ni and Cerwenka, 2016; Guan et al., 2021), thereby facilitating tumor progression. It is interesting to note that the above pathways related to M2 macrophage are activated in TCGA and GSE84437 datasets. These findings demonstrate that the overexpression of LOX promotes macrophage polarization towards M2 phenotype and activates a series of downstream signaling pathways to promote tumor progression.

Furthermore, M2 macrophage also contributes to chemoresistance (Dallavalasa et al., 2021). Fluorouracil, oxaliplatin, docetaxel, paclitaxel, and tegafur are usually used as the first-line chemotherapeutic drugs for gastric cancer (Wang et al., 2021). Methotrexate was considered as an effective strategy for peritoneal dissemination of gastric carcinoma (Imazawa et al., 2009). Our researches demonstrated that LOX expression has a negative correlation with the sensitivity of commonly used chemotherapeutic drugs for gastric cancer. M2 macrophage polarization induces tumor chemoresistance to paclitaxel and fluorouracil, which was well supported by many researches (Xing et al., 2021; Wei et al., 2019). Hence, we speculate that the overexpression of LOX may result in tumor chemoresistance of gastric cancer by promoting M2 macrophage polarization.

Research indicated that M2 macrophage can suppress T cell functions by expressing T cell immune checkpoint ligands, such as PD-L1 and PD-L2, to facilitate tumor immune escape (Wei et al., 2021). Our analysis suggested that LOX expression was positively associated with CTLA4, CD274 (PD-L1), HAVCR2, LAG3, PDCD1 (PD-1), PDCD1LG2 (PD-L2), and TIGIT. These results indicated that high LOX expression-mediated M2 macrophage polarization may be related to immune escape of cancer cells.

There were several limitations in this study. Firstly, the HIF1A-AS2/RP11-366L20.2-miRNA-29c axis was identified as one of the causes of high expression of LOX in gastric cancer, which needs to be verified by further molecular experiments. Secondly, RP11-65J21.3 was markedly decreased in STAD samples, and its low expression presented satisfactory prognosis, which seems contradictory. Expanding the sample size or performing a meta-analysis of expression and survival may clarify this contradiction.

**FIGURE 10:**
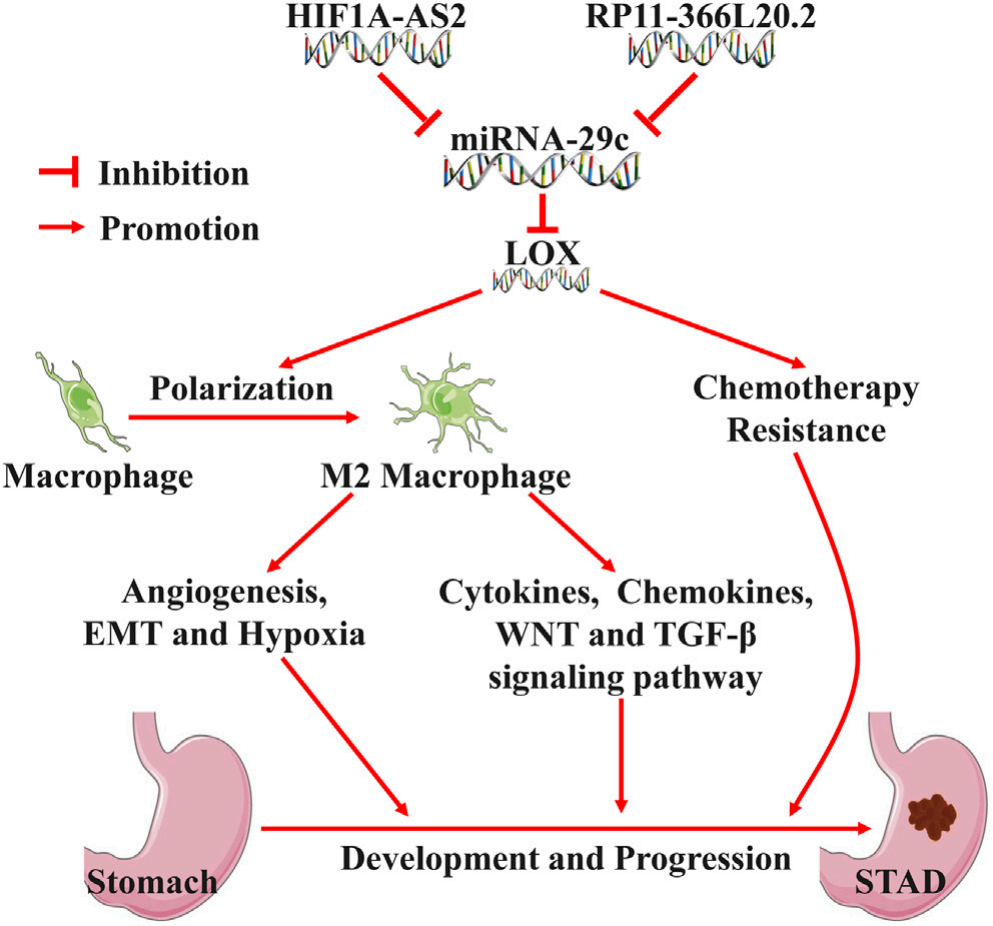
The underlying molecular mechanisms of HIF1A-AS2/RP11-366L20.2-miRNA-29c-mediated high expression of LOX in the carcinogenesis and development of gastric carcinoma.

In summary, our results demonstrate that the HIF1A-AS2/RP11-366L20.2-miRNA-29c axis-mediated high expression of LOX results in poor prognosis of gastric cancer by promoting M2 macrophage polarization and tumor immune escape and enhancing drug resistance of cancer cells to chemotherapeutic drugs (Figure 10). Targeting inhibition of LOX holds promise as a treatment strategy for gastric cancer.

## Data Availability

The datasets presented in this study can be found in online repositories. The names of the repository/repositories and accession number(s) can be found in the article/Supplementary Material.

## Supplementary Material

Supplementary Table S1 Six miRNA candidates that possessed LOX mRNA-binding sites.

Supplementary Table S2 Two hundred ninety-eight potential lncRNA candidates with miR-29c-3p binding sites

Supplementary Table S3 The 792 chemotherapeutic agents that received FDA approval or on clinical trials.
